# Virtual Sensor: Simultaneous State and Input Estimation for Nonlinear Interconnected Ground Vehicle System Dynamics

**DOI:** 10.3390/s23094236

**Published:** 2023-04-24

**Authors:** Chouki Sentouh, Majda Fouka, Jean-Christophe Popieul

**Affiliations:** 1 Université Polytechnique Hauts-de-France, LAMIH, CNRS, UMR 8201, F-59313 Valenciennes, France; majda.fouka@uphf.fr (M.F.); jean-christophe.popieul@uphf.fr (J.-C.P.); 2 INSA Hauts-de-France, F-59313 Valenciennes, France

**Keywords:** vehicle safety, vehicle dynamics, state estimation, unknown inputs estimation, interconnected observers, interlinked vehicle dynamics

## Abstract

This paper proposes a new observer approach used to simultaneously estimate both vehicle lateral and longitudinal nonlinear dynamics, as well as their unknown inputs. Based on cascade observers, this robust virtual sensor is able to more precisely estimate not only the vehicle state but also human driver external inputs and road attributes, including acceleration and brake pedal forces, steering torque, and road curvature. To overcome the observability and the interconnection issues related to the vehicle dynamics coupling characteristics, tire effort nonlinearities, and the tire–ground contact behavior during braking and acceleration, the linear-parameter-varying (LPV) interconnected unknown inputs observer (UIO) framework was used. This interconnection scheme of the proposed observer allows us to reduce the level of numerical complexity and conservatism. To deal with the nonlinearities related to the unmeasurable real-time variation in the vehicle longitudinal speed and tire slip velocities in front and rear wheels, the Takagi–Sugeno (T-S) fuzzy form was undertaken for the observer design. The input-to-state stability (ISS) of the estimation errors was exploited using Lyapunov stability arguments to allow for more relaxation and an additional robustness guarantee with respect to the disturbance term of unmeasurable nonlinearities. For the design of the LPV interconnected UIO, sufficient conditions of the ISS property were formulated as an optimization problem in terms of linear matrix inequalities (LMIs), which can be effectively solved with numerical solvers. Extensive experiments were carried out under various driving test scenarios, both in interactive simulations performed with the well-known Sherpa dynamic driving simulator, and then using the LAMIH Twingo vehicle prototype, in order to highlight the effectiveness and the validity of the proposed observer design.

## 1. Introduction

Autonomous driving and driver assistance systems are today the focus of several research works conducted both in public institutions and in industry. The motivation behind these research efforts and the massive investments related to driving automation is the potential benefits promised by this technology to improve road safety, provide mobility suitable for the elderly and disabled people, increase road capacity, save fuel, and reduce greenhouse gas emissions. Nevertheless, the complexity of vehicle models (coupling and nonlinear dynamics, parameter uncertainties, etc.) and the lack of knowledge of dynamic states and external inputs make the embeddability of advanced driver assistance systems (ADASs) more complex. All of these ADASs can be enhanced using real-time knowledge of the vehicle state evolution and unknown inputs, such as driver actions and road attributes. For example, vehicle stability and shared steering control systems require side slip angle and driver torque information for their control purposes [1,2,3,4], and future steer-by-wire systems, in which the mechanical steering of today will be replaced with either electrical, hydraulic, or electro-hydraulic steering, will require such information for full-state feedback shared control [5,6]. However, the vehicle state, as well as the inputs, are not all directly measurable: necessary sensors do not exist yet or are still too expensive for use in commercial vehicles. For example, current vehicles are not equipped with the ability to measure the side slip angle directly. With a cost of 20,000 EUR or more, the optical “Correvit” sensor is much too expensive for automotive applications. To solve this problem, virtual sensors based on estimation algorithms are usually used instead.

### 1.1. Related Works on Virtual Sensors

During the last few decades, a notable interest in designing virtual sensors (observers) using model-based estimation algorithms was demonstrated by a large body of literature for potential vehicle control and security applications in order to minimize the physical sensors’ cost [7,8,9]. Nonlinear estimation schemes were investigated to face nonlinear vehicle dynamics issues. A well-known method for estimating time-varying parameters of nonlinear models is the extended Kalman filter (EKF), adopted in [10] to overcome nonlinearities leading to performance deterioration. The vehicle longitudinal forces were reconstructed using an adaptive neural network nonlinear observer in [11]. A dual unscented Kalman filter algorithm, based on the nonlinear least-squares approach and the hybrid Levenberg–Marquardt, was used in [12] to estimate tire lateral and normal forces. A nonlinear observer for tracking vehicle motion trajectories on highways using a radar or laser sensor was addressed in [13]. Adaptive observers were introduced to study the convergence of state estimation jointly with system parameters identification [14,15]. The parameter estimation of nonlinear vehicle dynamics was investigated using a fuzzy unknown input observer (UIO) [16] and with a nonlinear adaptive observer [17].

When the estimation method is based on a physical vehicle model, the presence of unmodeled coupled dynamics, faults, or unknown inputs, which can be regarded as disturbances, can deteriorate the estimation. Different strategies have been investigated to simultaneously estimate vehicle dynamic states, external disturbances, or unknown inputs and faults. An LPV unknown inputs observer with Takagi–Sugeno representation formalized in the LMI framework was proposed in [18,19] for the estimation of both vehicle lateral dynamics and the driver’s steering torque. A simultaneous estimation of lateral dynamics and road attributes, including curvature, slope, and superelevation, was addressed in [20,21,22,23,24]. Steering and torque actuators’ faults detection was studied in [25], where the nonlinear vehicle dynamics were reformulated as an N-TS fuzzy form with both measured and unmeasured nonlinear outcomes in order to design a fault detector based on a nonlinear observer. However, only the lateral dynamics were estimated by the N-TS observer. In addition, it has already been pointed out that, for complex or large-scale systems, the limitations of the model-based observer concept are related to the complexity from a computation point of view for real-time implementation. The problem of finding the minimal representations for reducing the complexity and conservatism was studied by using different LPV representations [26], e.g., the linear fractional transformation (LFT) form, by investigating the polytopic descriptor form [27], etc. Recent research was conducted to study cascade systems or two-stage structures [28,29], which are very common configurations in engineering applications. The results reported in [30,31] for cascade systems reveal interesting results that provide parameters identification or a robust estimation of slow and fast dynamics variables. Particular attention was paid to the estimation of the tire–ground contact forces in [32] to improve vehicle safety using a delayed interconnected cascade–observer structure.

### 1.2. Proposed Methodology and Contributions

Most of the aforementioned papers assume that the observer design neglects tire–road contact efforts, or regard vehicle driving conditions as a small variation or a constant speed in order to have independent dynamics, which significantly simplifies the system design. Although a very interesting development from a theoretical point of view, this simplification is not an adequate representation of the real physical system when it is subjected to strong coupling dynamics, disturbances, and external unknown inputs. Despite extensive literature, the unknown input observer design for the *simultaneous* estimation of the vehicle longitudinal and lateral dynamics, the human driver actions, and the road attributes have not been well addressed. The effective integration of the interlinked vehicle observer presents several theoretical and technical challenges and very few works related to this topic can be found in the open literature. An interesting solution was proposed in our previous work in [33] for dealing with coupled vehicle lateral and longitudinal dynamics estimation using a quasi-LPV interconnected observer with hardware experiments performed with the well-known SHERPA dynamic car simulator under real-world driving situations. This version of the observer was extended in this paper by proposing a novel two-stage LPV interconnected unknown input observer (NI-UIO) for the estimation of the coupled and dependent lateral and longitudinal nonlinear vehicle motion together with tire–road interaction forces and unknown external inputs, namely driver traction and braking and steering torques, as well as the road curvature. More precisely, this estimation scheme has several merits:

- The main distinction of the proposed LPV estimation approach compared to the existing methods is that no decoupling of the vehicle interconnected dynamics nor nonlinearities considered as non-measurable time-varying external parameters are required for the reconstruction of both vehicle lateral and longitudinal nonlinear dynamics, as well as the unknown inputs. In particular, variations in the forward speed and tire slip velocities of the front and rear wheels are considered as unmeasurable nonlinearities in the interconnected scheme and processed through the boundary domain.
- The proposed interconnection configuration presents an interesting way to reduce the conservatism and give more relaxation for a complete vehicle observer design. This relaxation allows us to derive fewer linear matrix inequality (LMI) conditions for the optimization problem, which can be efficiently solved with numerical solvers.
- Based on the input-to-state stability property, the usual sign definition of the Lyapunov principle can be relaxed. It provides a framework in which we can formulate stability arguments with respect to input disturbances. Thus, it has the advantage of providing further theoretical guarantees of robustness against unknown inputs and disturbances, as well as non-measurable non-linearity terms.
- The effectiveness of the new interconnected configuration of the proposed UI observer algorithm was evaluated in a hardware interactive simulation on the “SHERPA full-scale car driving simulator” and then experimentally using the “Twingo” vehicle prototype platform, with a robustness test performed regarding road friction uncertainties.

The remainder of the paper is structured as follows. Section 2 describes the vehicle interlinked model with the tire–ground contact efforts. Section 3 presents this model through the interconnected T-S fuzzy model. Then, Section 4 illustrates the observer design and the convergence analysis based on the ISS-Lyapunov theory. Section 5 discusses the results obtained from both interactive simulations and real-world experiments. Finally, some concluding remarks with perspectives are given in the last section.

## 2. Interlinked Road–Vehicle Lateral and Longitudinal Dynamics

**Figure 1:**
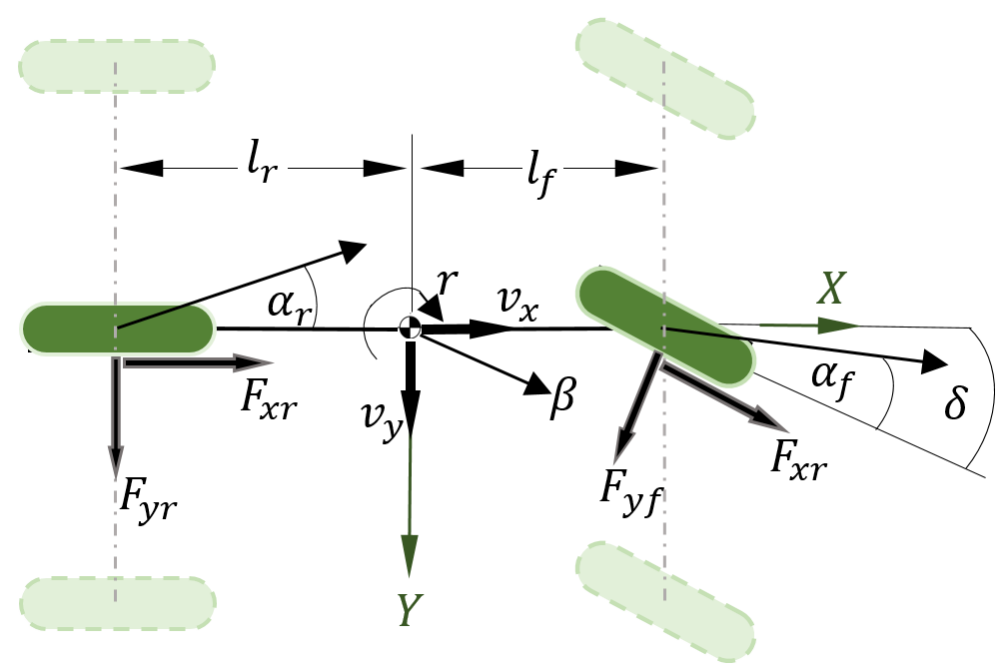
Nonlinear vehicle bicycle model.

Ground vehicles are complex systems with totally nonlinear and coupled dynamics that involve interlinked mechanical parts such as braking, suspension, steering, the powertrain, etc. The vehicle dynamics are described in the vehicle’s fixed frame with 12-DoF (twelve degrees of freedom), in which nonlinear longitudinal, lateral, and yaw motions, the vehicle steering system, and accelerator and brake pedals are considered with tire–ground forces, respectively. In addition, the vehicle positioning on the road is described via a standard vision dynamic model [34]. In the following, we describe the nonlinear model that captures the essential dynamics of the vehicle, developed under the assumption that the left and right wheels of each axle are grouped together to form a single equivalent tire, as shown in Figure 1, and the dynamics of the vertical, pitch, and roll movements are neglected [29].

- Longitudinal, lateral, and yaw motions:
  (1) $$\begin{array}{cc} m{\dot{v}}_{y} & =F_{xf}sin\left(\delta \right)+F_{yf}cos\left(\delta \right)+F_{yr}-mv_{x}r+F_{w}\\ m{\dot{v}}_{x} & =F_{xf}cos\left(\delta \right)-F_{yf}sin\left(\delta \right)+F_{xr}+mv_{y}r-F_{a}-F_{rr}\\ I_{z}\dot{r} & =l_{f}F_{yf}cos\left(\delta \right)-l_{r}F_{yr}+l_{f}F_{xf}sin\left(\delta \right)+l_{w}F_{w} \end{array}$$
- Wheels’ rotational movements:
  (2) $$\begin{array}{cc} I_{\omega_{f}}{\dot{\omega }}_{f} & =-RF_{xf}+T_{f}+B_{f}\\ I_{\omega_{r}}{\dot{\omega }}_{r} & =-RF_{xr}+B_{r} \end{array}$$
- Vehicle positioning on the road:
  (3) $$\begin{array}{cc} {\dot{y}}_{L} & =v_{y}+l_{s}r+\psi_{L}v_{x}\\ {\dot{\psi }}_{L} & =r-\kappa v_{x} \end{array}$$
- Electronic power steering system dynamics:
  (4) $$\ddot{\delta }=\frac{1}{R_{s}I_{s}}T_{s}-\frac{t_{s_{\beta }}}{R_{s}I_{s}}\delta +\frac{t_{s_{\beta }}}{R_{s}I_{s}}\frac{v_{y}}{v_{x}}+\frac{t_{s_{r}}}{R_{s}I_{s}}r-\frac{B_{s}}{I_{s}}\dot{\delta }$$

The main objective of considering a vision system is to estimate the road curvature κ, which will give us an additional degree of freedom in reconstructing the motion of the vehicle. Moreover, the lateral vehicle model is augmented with the steering system to estimate the total steering torque ($T_{s}$), which is composed of both the power assist torque and the driver torque. The driver torque can be easily reconstructed from the estimation of the total steering torque and the known assistance torque. $B_{f}$, $B_{r}$ are the braking torques applied to the front and rear tires, respectively, and $T_{B_{f}}=B_{f}+T_{f}$ is the total braking and traction torque, where $T_{f}$ is the engine torque applied on the front wheels. The vehicle parameters and variables are defined in the nomenclature section (see Appendix A). For small values of the tire side-slip angle α or slip velocity ratio λ, the lateral $F_{y}$ and longitudinal $F_{x}$ forces can be approximated by

(5) $$\frac{\sigma }{v_{x}}{\dot{F}}_{yi}=-F_{yi}+C_{\alpha_{i}}\alpha_{i}\;\mathrm{and}\;\frac{\sigma }{v_{x}}{\dot{F}}_{xi}=-F_{xi}+C_{\lambda_{i}}\lambda_{i}$$

σ is the tire relaxation that represents the transient time. The tire side slip angle α and the longitudinal tire slip ratio λ for the front and rear tires, respectively, are given as

(6) $$\begin{array}{cc} \; & \alpha_{f}=\delta -\frac{v_{y}+l_{f}r}{v_{x}}\;\mathrm{and}\;\alpha_{r}=-\frac{v_{y}-l_{r}r}{v_{x}}\\ \; & \lambda_{f}=\frac{\left(R\omega_{f}-v_{x}\right)}{max\{v_{x},R\omega_{f}\}}\;\mathrm{and}\;\lambda_{r}=\frac{\left(R\omega_{r}-v_{x}\right)}{max\{v_{x},R\omega_{r}\}} \end{array}$$

In order to consider the tire slip ratio during acceleration and braking, the following switching signal (denoted as $\varrho_{i}=\frac{1}{max\{v_{x},R\omega_{i}\}}$, i∈{r,f}) is considered:

(7) $$\varrho \left(t\right)=\left\{\begin{array}{ccc} \varrho_{i}=\frac{1}{\omega_{i}R}\; & \;\;\;\mathrm{if}\;\;\mathrm{Traction}: & \left\{\lambda_{i}>0,\;v_{x}<\omega_{i}R\right\}\\ \varrho_{i}=\frac{1}{v_{x}}\; & \;\;\;\mathrm{if}\;\;\mathrm{Braking}: & \left\{\lambda_{i}<0,\;v_{x}>\omega_{i}R\right\} \end{array}\right.$$

Then, $\lambda_{f}=\left(R\omega_{f}-v_{x}\right)\varrho_{f},\;\lambda_{r}=\left(R\omega_{r}-v_{x}\right)\varrho_{r}$. The variation in these nonlinear switched parameters $\varrho_{i}$ are treated as premise parameters and transformed into a T-S representation by the upper and lower bounds. In addition, we assume a small variation in the steering angle under normal driving conditions. In the next section, the T-S polytopic representation is undertaken using the well-known sector nonlinearity approach [26].

## 3. T-S Structure of the Interlinked Dynamics

Herein, the mathematical formulation for the time-varying interconnected system (1)–(5) leads to two-stage subsystems assembled in the interconnection scheme with three strong nonlinearities in each subsystem. This representation with its *q* varying parameters is exactly rewritten as a compact T-S form with the $r_{q}=2^{q}$ multi-model weighted by membership functions $\eta_{i}\left(\cdot \right)$ as follows:

(8) $$\left\{\begin{array}{c} \dot{X}=\underset{A_{\eta }}{\underset{︸}{\left[\begin{array}{cc} {\bar{A}}_{\eta } & 0\\ 0 & {\overset{˘}{A}}_{\eta } \end{array}\right]}}X+\underset{B_{\eta }}{\underset{︸}{\left[\begin{array}{cc} {\bar{B}}_{\eta } & 0\\ 0 & {\overset{˘}{B}}_{\eta } \end{array}\right]}}U+\underset{D_{\eta }}{\underset{︸}{\left[\begin{array}{cc} 0 & {\bar{D}}_{\eta }\\ {\overset{˘}{D}}_{\eta } & 0 \end{array}\right]}}\xi_{F}\\ Y=\underset{C}{\underset{︸}{\left[\begin{array}{cc} \bar{C} & 0\\ 0 & \overset{˘}{C} \end{array}\right]}}X \end{array}\right.$$

where $X\left(t\right)={\left[\bar{X}\left(t\right)\;\overset{˘}{X}\left(t\right)\right]}^{T}$ represents the state vector, with $\bar{X}\left(t\right)$ referring to $[v_{x},\omega_{f},\omega_{f},F_{xf},$$F_{xr}{]}^{T}$ for the longitudinal ($\sum_{x}$) and $\overset{˘}{X}\left(t\right)={\left[v_{y},r,\psi_{L},y_{L},F_{yf},F_{yr},\delta ,\dot{\delta }\right]}^{T}$ for the lateral ($\sum_{y}$) dynamics, $U\left(t\right)={\left[\bar{U}\left(t\right)\;\overset{˘}{U}\left(t\right)\right]}^{T}$ are the inputs of subsystems ($\sum_{x}$) and ($\sum_{y}$) with $\bar{U}={\left[T_{B_{f}},B_{r}\right]}^{T},\;\overset{˘}{U}={\left[T_{s},\kappa \right]}^{T}$, $Y\left(t\right)={\left[\bar{Y}\left(t\right)\;\overset{˘}{Y}\left(t\right)\right]}^{T}$ is the output vector with $\bar{Y}=\left[\omega_{f},\omega_{r},a_{x}\right],\;\overset{˘}{Y}={\left[r,\psi_{L},y_{L},a_{y},\delta \right]}^{T}$ the output vector for each subsystem, and $\xi_{F}\left(t\right)=\left[r_{y},F_{rr},F_{w}\right]$, $r_{y}=v_{y}r$. (${\bar{D}}_{\eta }$, ${\overset{˘}{D}}_{\eta }$) are the coupling matrices in the interconnection scheme. Therein, the nonlinearities considered here are related to tire slip velocities on the front and rear wheels $\varrho_{f}$, $\varrho_{r}$ and forward speeds $v_{x}$, $\frac{1}{v_{x}}$, $\frac{1}{v_{x}^{2}}$, considered as external immeasurable time-varying parameters. Let us consider that the time-varying matrices $\bar{\Pi }\in \left\{{\bar{A}}_{\eta },{\bar{B}}_{\eta },{\bar{C}}_{\eta },{\bar{D}}_{\eta }\right\}$ of the longitudinal subsystems and $\overset{˘}{\Pi }\in \left\{{\overset{˘}{A}}_{\eta },{\overset{˘}{B}}_{\eta },{\overset{˘}{C}}_{\eta },{\overset{˘}{D}}_{\eta }\right\}$ of lateral subsystems in (8) are continuous on the hypercube $\bar{\Theta },\overset{˘}{\Theta }$, with

(9) $${\bar{\Pi }}_{\eta }=\sum_{i=1}^{r_{q}}\eta_{i}\left(\bar{\theta }\right){\bar{\Pi }}_{i}\;\;\mathrm{and}\;\;{\overset{˘}{\Pi }}_{\eta }=\sum_{i=1}^{r_{q}}\eta_{i}\left(\overset{˘}{\theta }\right){\overset{˘}{\Pi }}_{i}\;r_{q}=2^{q}$$

where matrices ${\bar{\Pi }}_{i}$ and ${\overset{˘}{\Pi }}_{i}$ are constant for all $i\in [1,...,r_{q}]$. $r_{q}=2^{q}$ represents the number of local sub-models, where the *q* nonlinearities related to $\bar{\theta }\in \bar{\Theta }$, $\overset{˘}{\theta }\in \overset{˘}{\Theta }$ are captured via membership weighting functions $\eta_{i}\left(\cdot \right)$, which satisfy the convex-sum property in the compact set of the state space [26]

(10) $$\left\{\begin{array}{c} \sum_{i=1}^{r_{q}}\eta_{i}\left(\bar{\theta }\left(t\right)\right)=1,\;\sum_{i=1}^{r_{q}}{\dot{\eta }}_{i}=0,\;0\leq \eta_{i}\leq 1\\ \sum_{i=1}^{r_{q}}\eta_{i}\left(\overset{˘}{\theta }\left(t\right)\right)=1,\;\sum_{i=1}^{r_{q}}{\dot{\eta }}_{i}=0,\;0\leq \eta_{i}\leq 1\\ \forall i=\left\{1,2,..,r_{q}=2^{q}\right\} \end{array}\right.$$

where $\bar{\theta }\left(t\right)$ and $\overset{˘}{\theta }\left(t\right)$ are called the premise variables vector

(11) $$\theta :\left\{\begin{array}{c} \bar{\theta }\left(t\right)=\left\{v_{x},\;\;\varrho_{f},\;\;\varrho_{f}\right\}\;if\;\mathrm{Longitudinal}\;\mathrm{model}\\ \overset{˘}{\theta }\left(t\right)=\left\{v_{x},\;\;\frac{1}{v_{x}},\;\;\frac{1}{v_{x}^{2}}\right\}\;if\;\;\mathrm{Lateral}\;\mathrm{model} \end{array}\right.$$

The bounds of these smooth scheduling variables are defined in hyper-rectangles $\forall \bar{\theta }\in \bar{\Theta }$ and $\forall \overset{˘}{\theta }\in \overset{˘}{\Theta }$ given by

(12) $$\begin{array}{c} \bar{\Theta }:\left\{\bar{\theta }\in R^{q}\left|\begin{array}{c} {\bar{\theta }}_{i}^{\min}\leq {\bar{\theta }}_{i}\leq {\bar{\theta }}_{i}^{\max}:i=\left\{1,..,q\right\} \end{array}\right\}\right. \end{array}$$

(13) $$\begin{array}{c} \overset{˘}{\Theta }:\left\{\overset{˘}{\theta }\in R^{q}\left|\begin{array}{c} {\overset{˘}{\theta }}_{i}^{\min}\leq {\overset{˘}{\theta }}_{i}\leq {\overset{˘}{\theta }}_{i}^{\max}:i=\left\{1,..,q\right\} \end{array}\right\}\right. \end{array}$$

where ${\bar{\theta }}_{i}^{\min}$ and ${\bar{\theta }}_{i}^{\max}$ (respectively, ${\overset{˘}{\theta }}_{i}^{\min}$, ${\overset{˘}{\theta }}_{i}^{\max}$) are known lower and upper bounds on ${\bar{\theta }}_{i}$ (respectively, ${\overset{˘}{\theta }}_{i}$) for i={1,..,q}, and q=3 is the number of nonlinearities for each sub-model.

**Remark** **1.** *It was demonstrated in [27] that the descriptor structure can significantly reduce the LMIs conservativeness compared to the classical state space form. Note that the interconnected configuration (8) allows us to decrease the number of varying nonlinearities, which decreases the number of LMIs related to the induced sub-models. Consequently, the usual optimization problem is relaxed by exploiting the interconnected scheme, which leads to reducing the conservatism and computational complexity when solving the observer. The theoretical design allowing for this relaxation constitutes one of the main results of this paper.*

**Remark** **2.** *Note that an adequate choice of the nonlinearities used in the polytopic transformation allows for limiting the conservatism drawback, as stated in our previous works [35]. In this scope, the numerical complexity can be further reduced by exploiting the relation between the vehicle speed nonlinearities $v_{x}$, $\frac{1}{v_{x}}$ and $\frac{1}{v_{x}^{2}}$ using the first-element Taylor’s series simplification and a variable change as we proposed in our previous work [33].*

## 4. Observer Design

**Figure 2:**
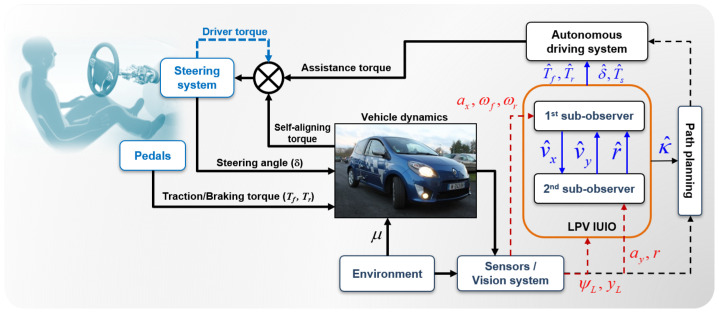
Architecture overview of the proposed interlinked UIO-based estimation approach.

The objective of this section is to design a two-stage nonlinear interconnected unknown input observer (NI-UIO) with state-dependent matrices and immeasurable nonlinearities. Therein, our analysis was conducted using the ISS-based Lyapunov function to guarantee the stability of the observer, whose dynamics depend on unknown disturbances or other inputs. An overall scheme of the system structure linked to the observer is depicted in Figure 2. To begin with, the following assumptions were considered.

**Assumption** **1.**

*(i)*  *The state ($\bar{x}$, $\overset{˘}{x}$) and the unknown inputs of the system are all bounded.*
*(ii)*  *The pairs $\left({\overset{˘}{A}}_{\eta },\overset{˘}{C}\right)$ and $\left({\bar{A}}_{\eta },\bar{C}\right)$ are observable or detectable in order to guarantee solutions to the LMI problem.*
  − *The polytopic sub-systems (8) are observable, i.e.,*
(14) $$\mathrm{rank}{\left[\begin{array}{c} C\;\;CA_{i}\;\cdots \;CA_{i}^{n-1} \end{array}\right]}^{T}=n\;\forall i\in \left[1,..,r_{q}\right]$$
  − *The polytopic LPV system (8) is detectable, i.e.,*
(15) $$\mathrm{rank}\left(\left[\begin{array}{cc} sI_{n}-A_{i} & B_{i}\\ C & 0 \end{array}\right]\right)=n+p\;s\in C$$
    *holds for all complex numbers s with Re(s)≥0 $X\left(t\right)\in R^{n}$, $U\left(t\right)\in R^{p}$.*
*(iii)*  *Each sub-observer exchanges some information through the interconnection scheme.*
*(iv)*  *The matching condition for the model holds*
(16) $$\mathrm{rank}\left(\bar{C}{\bar{B}}_{i}\right)=\mathrm{rank}\left({\bar{B}}_{i}\right),\;\mathrm{rank}\left(\overset{˘}{C}{\overset{˘}{B}}_{i}\right)=\mathrm{rank}\left({\overset{˘}{B}}_{i}\right)$$

Assumption (i) holds in open-loop and the vehicle remains in a bounded state-space region to guarantee stability. It is also assumed that, in manual operating mode, normal drivers can be expected to be capable of maintaining a stable vehicle motion. By assumption (iii), we mean that the estimator requests current state information from the neighboring subsystems through the interconnection because of the physical interactions of the vehicle motions. Assumptions (ii) and (iv) can easily be checked numerically.

### 4.1. NI-UIO Stability and Convergence Analysis

The NI-UIO design can be stated as follows:

(17) $$\left\{\begin{array}{c} \dot{Z}=\underset{N_{\eta }}{\underset{︸}{\left[\begin{array}{cc} {\bar{N}}_{\eta } & 0\\ 0 & {\overset{˘}{N}}_{\eta } \end{array}\right]}}Z+\underset{L_{\eta }}{\underset{︸}{\left[\begin{array}{cc} {\bar{L}}_{\eta } & 0\\ 0 & {\overset{˘}{L}}_{\eta } \end{array}\right]}}Y+\underset{G_{\eta }}{\underset{︸}{\left[\begin{array}{cc} 0 & {\bar{G}}_{\eta }\\ {\overset{˘}{G}}_{\eta } & 0 \end{array}\right]}}{\hat{\xi }}_{F}\\ \hat{X}=Z-\underset{H_{\eta }}{\underset{︸}{\left[\begin{array}{cc} \bar{H} & 0\\ 0 & \overset{˘}{H} \end{array}\right]}}Y \end{array}\right.$$

where $Z={\left[\bar{Z}\left(t\right)\;\overset{˘}{Z}\left(t\right)\right]}^{T}$ is the state of the observer, $\hat{X}\left(t\right)={\left[\hat{\bar{X}}\left(t\right)\;\hat{\overset{˘}{X}}\left(t\right)\right]}^{T}$ are the estimated states, and $Y\left(t\right)=[\bar{Y}\left(t\right)\;\overset{˘}{Y}\left(t\right)]$ are the output vectors. The observer gains $N_{\eta }$, $G_{\eta }$, $L_{\eta }$, and $H_{\eta }$ are written as (9). In the following, the observer design procedure aims to determine the aforementioned observer’s matrices. Let us consider the following suitable state estimation error:

(18) $$\begin{array}{ccc} e(t) & = & X\left(t\right)-\hat{X}\left(t\right)=T_{\eta }\;X\left(t\right)-Z\left(t\right)\\ \mathrm{with}\;\;T_{\eta } & = & \sum_{i=1}^{r_{q}}\eta_{i}\left(\theta \right)T_{i}\;\;\mathrm{and}\;\;T_{i}=I+H_{i}\;C \end{array}$$

According to the observer (17) and the system Equation (8), the dynamics of the estimation error is given as

(19) $$\begin{array}{ccc} \dot{e} & = & {\dot{T}}_{\eta }X+T_{\eta }\;\dot{X}-\dot{Z}\\ & = & {\dot{T}}_{\eta }X+T_{\eta }\left(A_{\eta }X+B_{\eta }\;U+D_{\eta }\;\xi_{F}\right)-\left(N_{\eta }Z+G_{\eta }\;{\hat{\xi }}_{F}+L_{\eta }\;Y\right)\\ & = & \left[\begin{array}{cc} {\bar{N}}_{\eta } & 0\\ 0 & {\overset{˘}{N}}_{\eta } \end{array}\right]e+\left[\begin{array}{cc} {\bar{\Phi }}_{\eta } & 0\\ 0 & {\overset{˘}{\Phi }}_{\eta } \end{array}\right]x+\left[\begin{array}{cc} {\bar{T}}_{\eta }{\bar{B}}_{\eta } & 0\\ 0 & {\overset{˘}{T}}_{\eta }{\overset{˘}{B}}_{\eta } \end{array}\right]U\\ & + & \left[\begin{array}{cc} {\bar{G}}_{\eta } & 0\\ 0 & {\overset{˘}{G}}_{\eta } \end{array}\right]\Delta_{\xi }+\left[\begin{array}{cc} {\bar{T}}_{\eta }{\bar{D}}_{\eta }-{\bar{G}}_{\eta } & 0\\ 0 & {\overset{˘}{T}}_{\eta }{\overset{˘}{D}}_{\eta }-{\overset{˘}{G}}_{\eta } \end{array}\right]\xi_{F} \end{array}$$

(20) $$\begin{array}{cc} \;\mathrm{with} & \;{\bar{\Phi }}_{\eta }={\bar{T}}_{\eta }\;{\bar{A}}_{\eta }+{\dot{\bar{T}}}_{\eta }-{\bar{L}}_{\eta }\;\bar{C}-{\bar{N}}_{\eta }{\bar{T}}_{\eta }\\ & {\overset{˘}{\Phi }}_{\eta }={\overset{˘}{T}}_{\eta }\;{\overset{˘}{A}}_{\eta }+{\dot{\overset{˘}{T}}}_{\eta }-{\overset{˘}{L}}_{\eta }\;\overset{˘}{C}-{\overset{˘}{N}}_{\eta }{\overset{˘}{T}}_{\eta },\;\Delta_{\xi }=\xi_{F}-{\hat{\xi }}_{F} \end{array}$$

In order to satisfy the stability of the error dynamics (19), the following conditions must be guaranteed:

(21) $$\begin{array}{ccc} & & {\bar{\Phi }}_{\eta }=0,\;{\overset{˘}{\Phi }}_{\eta }=0 \end{array}$$

(22) $$\begin{array}{ccc} & & {\bar{T}}_{\eta }{\bar{B}}_{\eta }=0,\;{\overset{˘}{T}}_{\eta }{\overset{˘}{B}}_{\eta }=0 \end{array}$$

(23) $$\begin{array}{ccc} & & {\bar{T}}_{\eta }{\bar{D}}_{\eta }-{\bar{G}}_{\eta }=0,\;{\overset{˘}{T}}_{\eta }{\overset{˘}{D}}_{\eta }-{\overset{˘}{G}}_{\eta }=0 \end{array}$$

Consequently, the estimation error dynamics e(t) become

(24) $$\begin{array}{ccc} \dot{e} & = & \left[\begin{array}{cc} {\bar{N}}_{\eta } & 0\\ 0 & {\overset{˘}{N}}_{\eta } \end{array}\right]\times e+\left[\begin{array}{cc} {\bar{G}}_{\eta } & 0\\ 0 & {\overset{˘}{G}}_{\eta } \end{array}\right]\;\Delta_{\xi } \end{array}$$

- e(t)→0 asymptotically if $\Delta_{\xi }\left(t\right)=0$;
- Bounded error e(t) if $\Delta_{\xi }\left(t\right)\neq 0$.

This is a fundamental prerequisite for the main ISS analysis to verify the impact of perturbation on the asymptotic bound of the solutions. The following steps in the design approach are followed to satisfy the stability of the error dynamics: (24)

(1) Condition (21) allows us to compute the Hurwitz gains
  (25) $$\begin{array}{cc} & {\bar{N}}_{\eta }={\bar{\Gamma }}_{\eta }-{\bar{K}}_{\eta }\bar{C},\;{\overset{˘}{N}}_{\eta }={\overset{˘}{\Gamma }}_{\eta }-{\overset{˘}{K}}_{\eta }\overset{˘}{C}\\ \mathrm{With} & \begin{array}{cc} {\bar{\Gamma }}_{\eta } & ={\bar{T}}_{\eta }{\bar{A}}_{\eta }+{\dot{\bar{T}}}_{\eta },\;{\overset{˘}{\Gamma }}_{\eta }={\overset{˘}{T}}_{\eta }{\overset{˘}{A}}_{\eta }+{\dot{\overset{˘}{T}}}_{\eta },\\ {\bar{K}}_{\eta } & ={\bar{N}}_{\eta }\;{\bar{H}}_{\eta }+{\bar{L}}_{\eta },\;{\overset{˘}{K}}_{\eta }={\overset{˘}{N}}_{\eta }\;{\overset{˘}{H}}_{\eta }+{\overset{˘}{L}}_{\eta } \end{array} \end{array}$$
(2) In order to make the state estimation error independent of the UI, the equality constraint (22) can be equivalently written as the decoupling condition in (16). This leads us to find matrices $H_{\eta }$, i.e., (${\bar{H}}_{\eta },{\overset{˘}{H}}_{\eta }$)
  (26) $$\begin{array}{ccc} T_{\eta }B_{\eta }=0 & \Leftrightarrow & \left(I_{n}+H_{\eta }C\right)B_{\eta }=0\\ {\bar{H}}_{\eta }=-{\bar{B}}_{\eta }{\left(\bar{C}{\bar{B}}_{\eta }\right)}^{†} & & {\overset{˘}{H}}_{\eta }=-{\overset{˘}{B}}_{\eta }{\left(\overset{˘}{C}{\overset{˘}{B}}_{\eta }\right)}^{†} \end{array}$$
  where ${\left(\cdot \right)}^{†}={\left({\left(\cdot \right)}^{T}\left(\cdot \right)\right)}^{-1}{\left(\cdot \right)}^{T}$ is the left pseudo-inverse of (·).
(3) After computing $H_{\eta }$, we obtain: $T_{\eta }=I_{n}+H_{\eta }C_{\eta }$; then, from (23), $G_{\eta }=T_{\eta }D_{\eta }$.

The following theorem 1 states the main result in terms of LMIs ensuring the ISS convergence of the state vector.

**Theorem** **1.** *In view of the two-stage longitudinal and lateral subsystems subject to unknown inputs, if the polytopic interlinked models (8) satisfy the stated Assumptions 1, an NI-UIO observer is designed by (17), and the ISS convergence of the estimation errors is ensured, then the origin of the system will be practically finite-time stable, i.e., the system states will converge to the neighborhood of the origin in finite time.*

*Step 1: Give the varying parameter-dependent matrices (${\bar{N}}_{\eta }$ and ${\overset{˘}{N}}_{\eta }$), (${\bar{L}}_{\eta }$ and ${\overset{˘}{L}}_{\eta }$), (${\bar{G}}_{\eta }$ and ${\overset{˘}{G}}_{\eta }$), and (${\bar{H}}_{\eta }$ and ${\overset{˘}{H}}_{\eta }$).*

*Step 2: For given real positive scalars α, a, and matrices $G=\{\bar{G},\overset{˘}{G}\}$, if there exist two symmetric positive definite matrices $\bar{P}$ and $\overset{˘}{P}$, and gains matrices ${\bar{\Omega }}_{i}$ and ${\overset{˘}{\Omega }}_{i},\;\;i=1,...,r_{q}$, positive scalars $\eta =diag\{\eta_{1},\eta_{2}\}$ are the solutions of the following LMI optimization problem:*

(27a) $$\min_{\bar{P},\overset{˘}{P},\eta_{1},\eta_{2}}\;a\eta_{1}+\left(1-a\right)\eta_{2}\;a\in \left[0,1\right]$$

(27b) $$\left[\begin{array}{cc} {\bar{\Gamma }}_{i}^{T}\bar{P}+\bar{P}{\bar{\Gamma }}_{i}-{\bar{C}}^{T}{\bar{\Omega }}^{T}-\bar{\Omega }\bar{C}+\alpha \bar{P} & \bar{P}{\bar{G}}_{i}\\ {\bar{G}}_{\eta }^{T}\bar{P} & -{\bar{G}}^{-1} \end{array}\right]<0$$

(27c) $$\left[\begin{array}{cc} {\overset{˘}{\Gamma }}_{i}^{T}\overset{˘}{P}+\overset{˘}{P}{\overset{˘}{\Gamma }}_{i}-{\overset{˘}{C}}^{T}{\overset{˘}{\Omega }}^{T}-\overset{˘}{\Omega }\overset{˘}{C}+\alpha \overset{˘}{P} & \overset{˘}{P}{\overset{˘}{G}}_{i}\\ {\overset{˘}{G}}_{\eta }^{T}\overset{˘}{P} & -{\overset{˘}{G}}^{-1} \end{array}\right]<0$$

(27d) $$\left[\begin{array}{cc} \alpha \eta_{1}I_{n} & \bar{P}\\ \bar{P} & \alpha \eta_{1}I_{n} \end{array}\right]>0,\left[\begin{array}{cc} \alpha \eta_{2}I_{n} & \overset{˘}{P}\\ \overset{˘}{P} & \alpha \eta_{2}I_{n} \end{array}\right]>0$$

(27e) $$\;\bar{P}\geq I_{n}\;\overset{˘}{P}\geq I_{n}$$

*Step 3: The observer gains are given by*

(28) $$\begin{array}{cc} {\bar{K}}_{\eta } & ={\bar{P}}_{\eta }^{-1}{\bar{\Omega }}_{\eta },\;{\bar{N}}_{\eta }={\bar{\Gamma }}_{\eta }-{\bar{K}}_{\eta }\bar{C},\;{\bar{L}}_{\eta }={\bar{K}}_{\eta }-{\bar{N}}_{\eta }\bar{H}\\ {\overset{˘}{K}}_{\eta } & ={\overset{˘}{P}}_{\eta }^{-1}{\overset{˘}{\Omega }}_{\eta },\;{\overset{˘}{N}}_{\eta }={\overset{˘}{\Gamma }}_{\eta }-{\overset{˘}{K}}_{\eta }\overset{˘}{C},\;{\overset{˘}{L}}_{\eta }={\overset{˘}{K}}_{\eta }-{\overset{˘}{N}}_{\eta }\overset{˘}{H} \end{array}$$

### 4.2. Algebraic Reconstruction of Unknown Inputs

In this section, we address the unknown input reconstruction of the vehicle’s longitudinal and lateral dynamics. We focus our interest on the front and rear braking and traction torques, the steering torque, and the road curvature, since they play a key role in guaranteeing vehicle stability in driving maneuvers. In order to avoid the direct use of the output derivative, we first consider a high-order sliding mode differentiator that can provide an exact estimation of the output derivatives [36]. From the vehicle dynamics (8) and $\hat{Y}=C\hat{X}\left(t\right)$, we obtain

(29) $$\dot{Y}\left(t\right)=CA_{\eta }\hat{X}\left(t\right)+CB_{\eta }U\left(t\right)+CD_{\eta }{\hat{\xi }}_{F}$$

From the design of the derivatives estimates $\dot{\hat{Y}}$ obtained from the high-order sliding mode differentiator and the states estimate $\hat{X}$, the unknown inputs $\hat{U}$ can be reconstructed by an algebraic inversion of the previous equation under the fulfilled rank condition $rank\left(CB_{\eta }\right)=rank\left(B_{\eta }\right)$

(30) $$\hat{U}:\left\{\begin{array}{c} \hat{\bar{U}}={\left(\bar{C}{\bar{B}}_{\eta }\right)}^{†}\left(\dot{\bar{Y}}-\bar{C}{\bar{A}}_{\eta }\hat{\bar{X}}\left(t\right)-\bar{C}{\bar{D}}_{\eta }{\hat{\bar{\xi }}}_{F}\right)\\ \hat{\overset{˘}{U}}={\left(\overset{˘}{C}{\overset{˘}{B}}_{\eta }\right)}^{†}\left(\dot{\overset{˘}{Y}}-\overset{˘}{C}{\overset{˘}{A}}_{\eta }\hat{\overset{˘}{X}}\left(t\right)-\overset{˘}{C}{\overset{˘}{D}}_{\eta }{\hat{\overset{˘}{\xi }}}_{F}\right) \end{array}\right.$$

On the other hand, the convergence of $\hat{U}$ toward U can be analyzed by defining the unknown part estimation error and replacing $\Delta_{\xi }=G_{\eta }^{†}\left(\dot{e}-N_{\eta }e\right)$ as

(31) $$e_{U}=U-\hat{U}=-{\left(CB_{\eta }\right)}^{†}\left(CA_{\eta }e+CD_{\eta }G_{\eta }^{†}\left(\dot{e}-N_{\eta }e\right)\right)$$

e(t) satisfy the ISS performance; then, the unknown inputs converge toward a small region to achieve the ISS property.

## 5. Experimental Results and Discussions

### 5.1. Hardware Experiments

**Figure 3:**
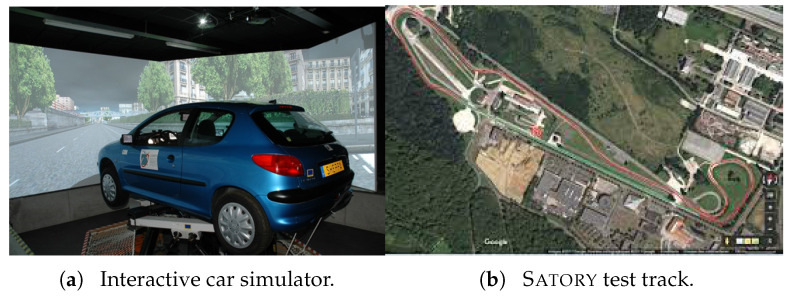
LamihSherpa car driving simulator.

The NI-UIO performance was validated first using hardware experiments through a series of driving maneuvers conducted with a human driver in the Sherpa–Lamih dynamic driving simulator. This interactive car simulator reproduces the vehicle dynamics taking into account a wide variety of parameters, such as weather conditions, grip, and the road surface [35]. It includes a full car mock-up Peugeot 206 vehicle installed on a six-DoF Stewart platform, presented in Figure 3a.

**Figure 4:**
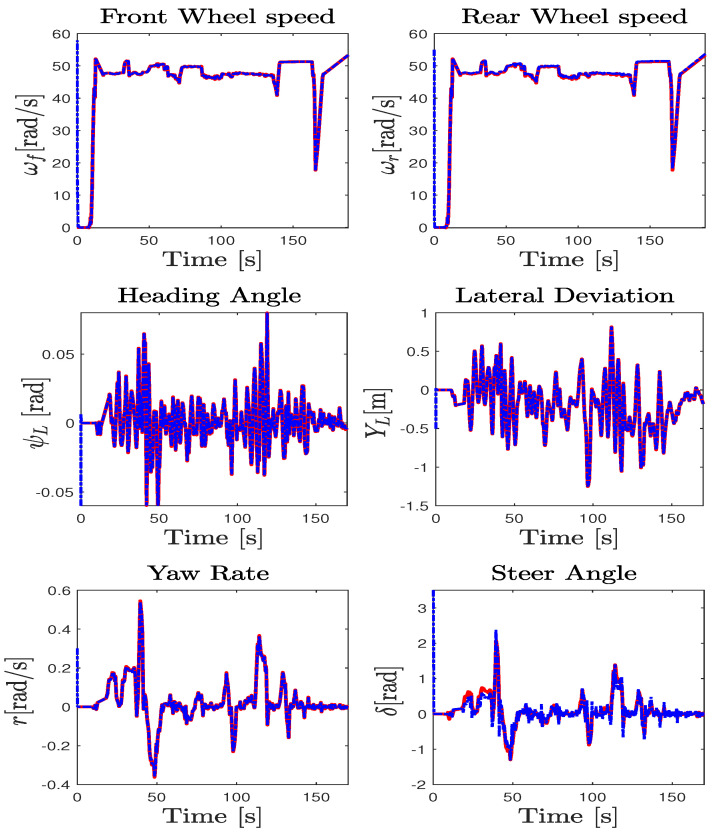
Sherpa car driving simulator data (solid red line) and estimation (dashed blue line).

**Figure 5:**
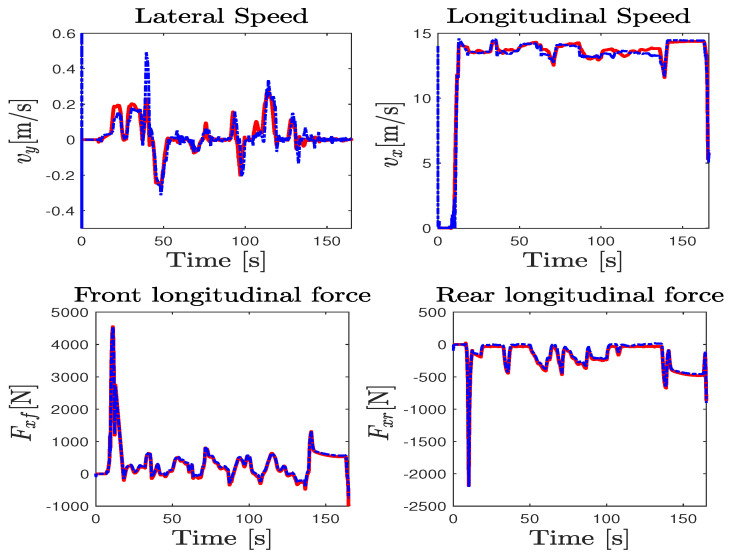
Longitudinal tire forces and velocities estimation performance: Sherpa car driving simulator (solid red line) and observer (dashed blue line).

**Figure 6:**
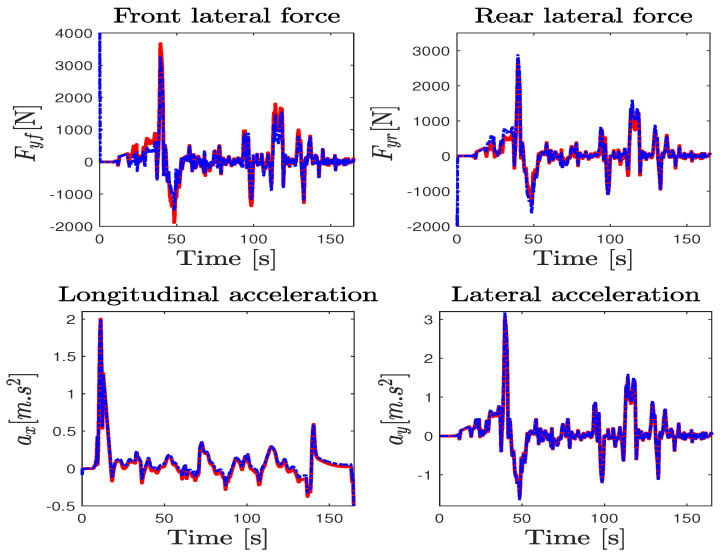
Lateral tire forces and accelerations estimation performance: Sherpa car driving simulator (solid red line) and observer (dashed blue line).

The test maneuver was performed on the Satory test track considering a dry asphalt road with the maximum mobilizable friction coefficient fixed at μ=1. This test track as presented in Figure 3b is composed of straight lines followed by several narrow and big bend profiles. It is very interesting to evaluate the proposed observer and the ISS performance of this path trajectory configuration since we can test a wide spectrum of the vehicle dynamics under and over its linearization interval. The data were collected with a sampling time of 0.01 s from the simulator and the observer was implemented to work with the same frequency. The estimation of the wheels’ angular velocities, yaw rate, and steering angle, as well as the vehicle positioning on the road defined by the lateral deviation and the heading errors, provided by the NI-UIO using their counterpart measured vehicle data coming from the driving simulator, are depicted in Figure 4. Since these signals are measured and used in the observer design, the state estimation results of Figure 4 demonstrate a finite-time estimation convergence. Hence, Figure 5 and Figure 6 depict the estimation results of unmeasured state variables, namely the lateral and forward speeds $v_{y},v_{x}$, the front/rear lateral tire forces $F_{yf},F_{yr}$, and the front/rear longitudinal tire forces $F_{xf},F_{xr}$. Comparing the estimated states with those provided by the car dynamic driving simulator, we can see that the observer has a fast dynamic transition and a good estimation convergence.

**Figure 7:**
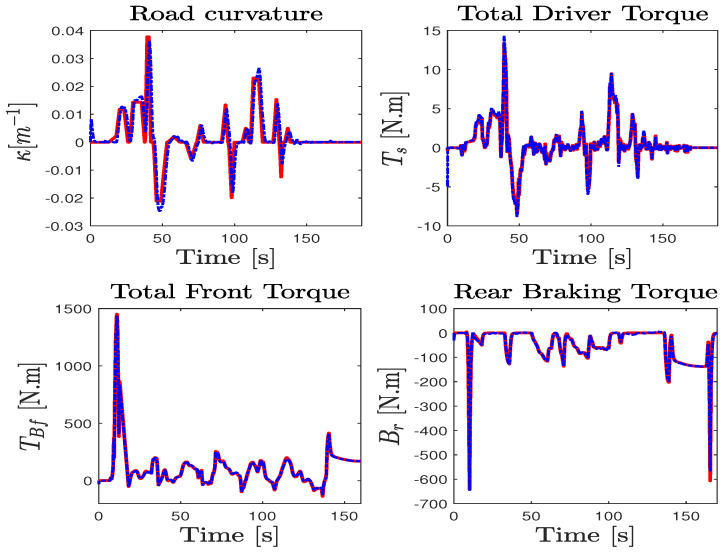
Unknown input estimation performance: Sherpa (solid red line) and observer (dashed blue line).

For a more faithful validation, the unmeasured states ($F_{x_{i}}$, $F_{y_{i}}$) were used to reconstruct the lateral and longitudinal accelerations $a_{y},a_{x}$ given by $m{\hat{a}}_{y}=\sum {\hat{F}}_{yi}$ and $m{\hat{a}}_{x}=\sum {\hat{F}}_{xi}$, where i={f,r}. It is obvious that the results reported in Figure 6 show a finite-time asymptotic estimation even for a coupled driving maneuver. On the other hand, the unknown inputs, namely the two braking and accelerating torques on both front/rear wheels applied to manage the forward speed and the total steering torque applied on the lateral model, are well estimated from the model inversion together with the road curvature depicted in Figure 7 compared to nominal values obtained from the simulator. According to these results, it can be appreciated that the observer provides a good estimation accuracy under highly dynamic maneuvering, and proves the effectiveness of the approach in simultaneously estimating the dynamic states and the unknown inputs with ISS performances.

### 5.2. Observer Sensitivity against Road Friction Uncertainties

It is important to note that the observer was designed for a nominal case with road friction coefficient μ=1 (dry asphalt). To assess the observer sensitivity to the road uncertainties, the observer was tested with respect to the friction coefficient variation. To this end, two cases (moderately wet road μ=0.6 and very wet road μ=0.4) for the same digital database of the Satory test track were considered and compared with the nominal case by means of the root-mean-square errors (${RMSE}_{\%}$) and normalized mean-square errors (${NMSE}_{\%}$) considering the difference between the estimated and measured states and UI presented in Table 1. The metrics used in Table 1 are defined as

(32) $$\begin{array}{cc} \; & {NMSE}_{\%}=100-\frac{100({∥y-\hat{y}∥}^{2})}{{∥y-mean(y)∥}^{2}},{RMSE}_{\%}=100\sqrt{\frac{1}{N}\sum_{i=1}^{N}{\left(y-\hat{y}\right)}^{2}} \end{array}$$

where ‖‖ indicates the 2-norm of a vector and $\hat{y}$ is the estimate of *y* of length *N*. The errors must not contain any NaN or Inf values. We omitted the large picks in the computed metrics. From Table 1, the observer gives the better estimation for the nominal case where μ=1, where the maximal values of (${RMSE}_{\%}$) are the lowest and ${NMSE}_{\%}$ the largest. As expected, the estimation errors increase when the road friction decreases, with a maximum ${RMSE}_{\%}$ degradation of (3%). Moreover, the amplitudes of the deviation errors are more notable for the torques ($T_{Bf},B_{r},T_{s}$) estimations. Otherwise, it can be seen that the ${RMSE}_{\%}$ for the yaw rate and curvature remains approximately constant, so the observer is more robust against the friction parameter uncertainty. Indeed, even with road uncertainties, the deviation amplitude is quantified with ${RMSE}_{\%}<9.18\%$ and ${NMSE}_{\%}>84.61\%$. From the quantification result, we note that the observer still has good ISS performances in limiting the effect of the road grip variation on the vehicle state estimation.

### 5.3. Experiment Validation Procedure and Trials

These experimental log-data principally aim to point out the performance of the proposed NI-UIO in real-world driving situations and to show that the observer fulfills the unknown part reconstruction, which is one of the contributions of this paper.

**Figure 8:**
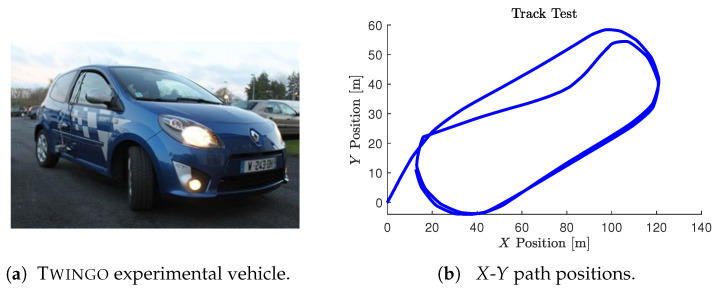
Lamih experimental test track.

The experiments were performed using the Lamih Renault Twingo experimental vehicle prototype depicted in Figure 8. This test bench encloses an embedded computer interfaced with various sensors and actuators used to measure the vehicle’s lateral and longitudinal dynamics. The data were collected with a sampling time of 0.01 s from the sensors and transmitted to the vehicle through the CAN bus. The experimental vehicle is equipped with a MicroAutobox unit from dSPACE for actuation purposes. Moreover, the platform is fully equipped with a Correvit sensor that measures the side slip angle and lateral speed, installed on the right back door at a height of 40 cm. The onboard acquisition system also includes a six-degrees-of-freedom inertial measurement unit (IMU) placed near the center of gravity to provide the acceleration, the three Euler angles, and their associated angular velocities in the three directions. The camera and GPS can record the scenario and the test path, respectively. The front-wheel steering angle was obtained from an optical encoder, whereas the angular speed of the wheels was directly obtained from the ABS sensors of each wheel.

### 5.4. Vehicle Model Adequacy Evaluation

**Figure 9:**
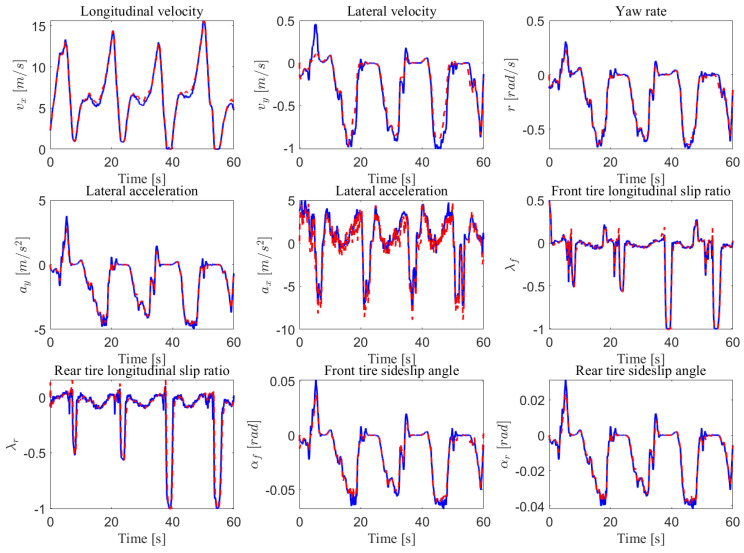
Vehicle model validation: measured data (solid blue line), model data (dashed red line).

The parameters of the road–vehicle model (1)–(5) describing the interconnected longitudinal and lateral vehicle dynamics were obtained from an identification process using recorded experimental data. Figure 9 compares the experimental data and the simulation results obtained from the model. Consequently, Table 2 summarizes the different computed metrics characterizing the model fit in percentage by means of the normalized values of the mean-square errors (NMSEs), the normalized root-mean-square errors (RMSEs), and the normalized mean errors (MEANs), considering the difference between the model outputs and the measured one. The variable states obtained from the vehicle model have a normalized RMSE approximately lower than 5%. Moreover, the comparison of the tire forces obtained from the model and those calculated from the measured data reveals a normalized RMSE lower than 10%. It can be seen from Figure 9 and Table 2 that the simulation results are quite good and that they are near the experiment ones, which demonstrates the ability of the model used to reproduce the dynamic behavior of the vehicle. Table A1 summarizes the parameters values of the LAMIH Renault TWINGO experimental vehicle prototype.

### 5.5. NI-UI Observer Validation

**Figure 10:**
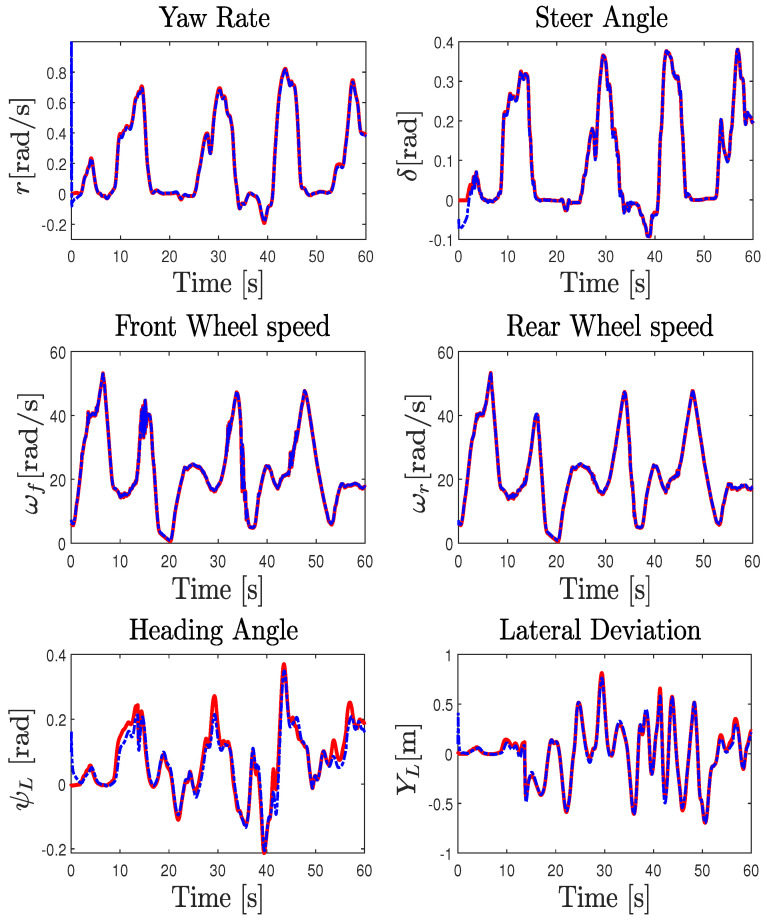
Experimental test results: Real measurements (solid red line) and observer estimation (dashed blue line).

**Figure 11:**
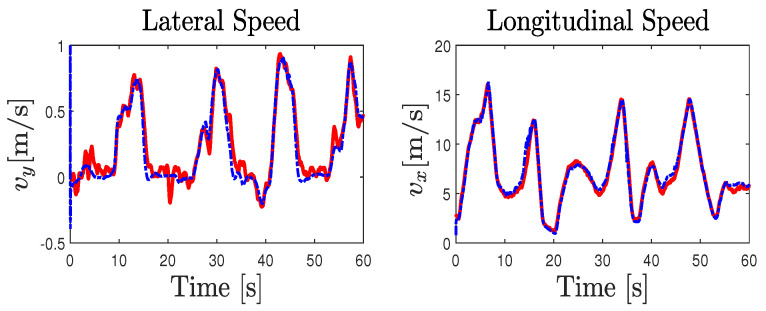
Experimental test results: Correvit speeds measurements (solid red line) and observer estimation (dashed blue line).

**Figure 12:**
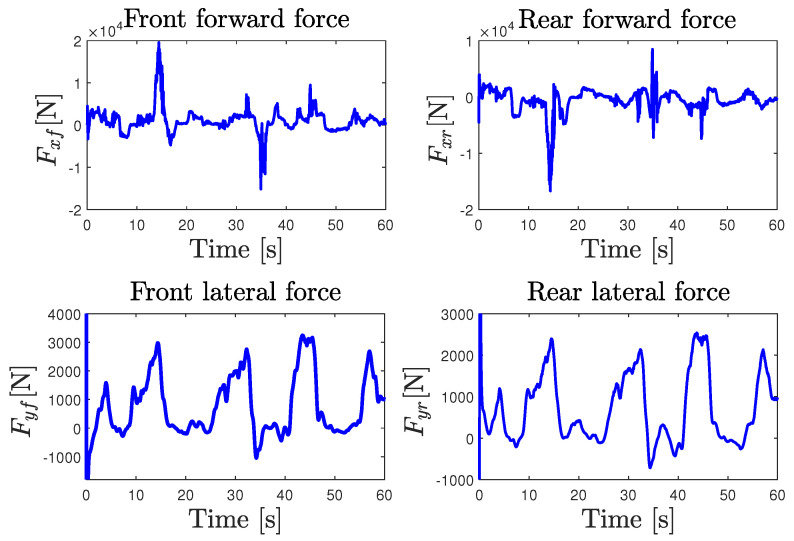
Experimental test results: longitudinal and lateral forces estimation.

**Figure 13:**
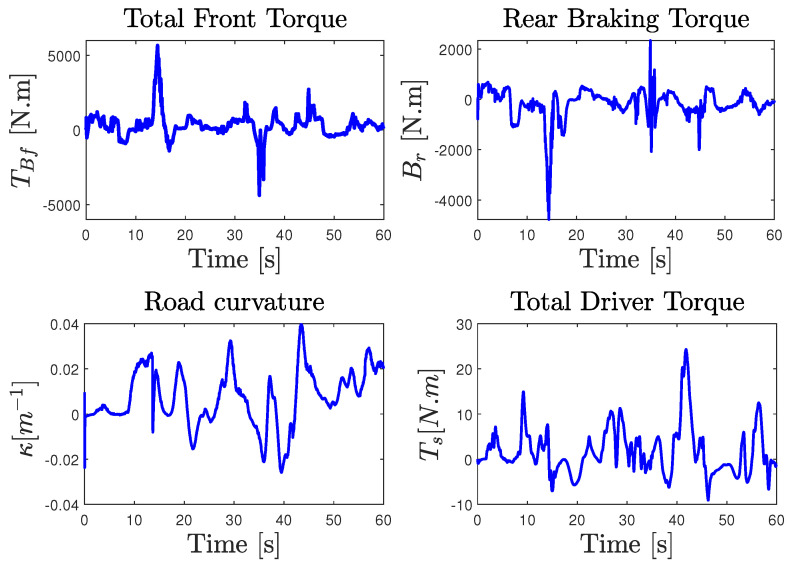
Experimental test results: Unknown input reconstruction.

The validity of the NI-UI observer was investigated on an urban dry road depicted in Figure 8. In this scenario, we considered a variable and a rapid change in the longitudinal speed of the vehicle with different driving conditions, including intensive braking and a high coupling of the longitudinal and lateral dynamics. The experimental data provided by the IMU sensor coupled with a dual GPS, including an RTK (real-time kinematic) base station used to improve the positional accuracy, were processed by a fusion system. All of the measured variables were sampled at 0.01s. The comparison of Figure 10 shows that the observer gives a good estimation of the measured variables used in the estimation algorithm. It should be noted that, during the experimental maneuver, the true torque inputs and the curvature are unknown and immeasurable. The state estimation results are presented in Figure 11 and Figure 12. Braking, traction, and driver steering torques, as well as the curvature, were reconstructed from the inversion method and are plotted in Figure 13.

**Figure 14:**
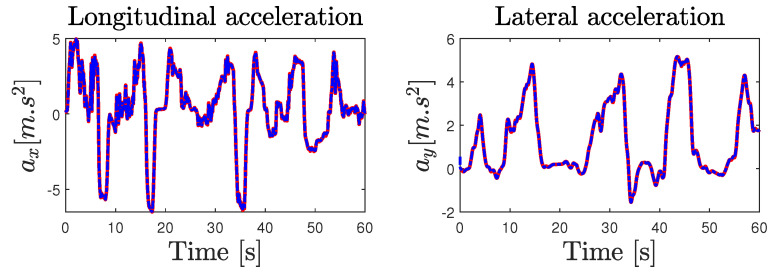
Experimental test results: acceleration estimation through tire force estimates.

**Figure 15:**
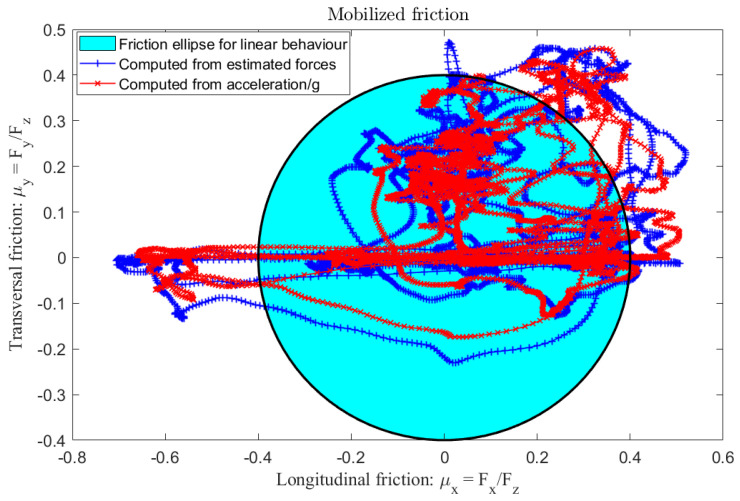
Experimental test results: longitudinal and transversal mobilized friction.

Note that the lateral and longitudinal speeds provided by the high-precision Correvit sensor serve only for observer validation and were not used in the design process. The performances of the lateral and longitudinal forces estimation were compared with the one measured by the IMU sensor through the accelerations, as shown in Figure 14. The experimental results illustrate that the observer quickly and accurately estimates the states with minimal error. The mobilized friction in longitudinal and lateral directions, computed from the force estimates obtained by the nonlinear NI-UI observer, was compared to the normalized acceleration ($a_{x}/g,\;a_{y}/g$) measured by the IMU sensor and is plotted in Figure 15. It can be seen from this figure that the conditions of the experimental test greatly exceed the linear domain of the tire forces evolution, represented by the friction ellipse with cyan color. Moreover, the proposed nonlinear observer is able to reconstruct the nonlinear dynamics of the vehicle even under heavy braking and coupled longitudinal and lateral dynamics conditions. Hence, the ISS performances are guaranteed and the estimation is acceptable under high deceleration and soft acceleration, as we can see in Figure 15. Finally, the interest in using a nonlinear NI-UIO estimation with immeasurable nonlinearities was validated with two test benches against road friction uncertainties and for different levels of acceleration and braking to evaluate the observer sensitivities. In particular, the immeasurable switching signal of Equation (7) used to represent the tire slip ratio during acceleration and braking is a very interesting contribution.

## 6. Conclusions

This paper presented a novel LMI-based virtual sensor for a simultaneous state and input estimation of nonlinear interconnected vehicle dynamics. In order to deal with nonlinearities related to the unmeasurable real-time variation in the vehicle’s longitudinal speed and tire slip velocities in front and rear wheels, and to overcome the interconnection issues, the vehicle model was first represented through a polytopic LPV interconnected T-S fuzzy model, and then the LPV interconnected unknown inputs observer framework was investigated. In particular, the interconnection scheme of the proposed observer was exploited to reduce the level of numerical complexity for the practical applicability of the virtual sensor. The proposed observer gives a very promising solution because it is capable of more precisely estimating not only the vehicle state, but also human driver external inputs and road attributes, including acceleration and brake pedal forces, steering torque, and road curvature, whose necessary sensors are very expensive. Another technical solution proposed in this paper is the estimation of the tire’s forces, which are very hard to measure with physical sensors. Moreover, the interconnection structure of the observer allows for the relaxation of the mutual dependence and coupling between the longitudinal and lateral motion, and thus reduces conservatism and the computational complexity.

Based on the ISS property, the stability and robustness of the proposed unknown input observer against unknown inputs and disturbances terms are guaranteed, taking into account real constraints such as the variations in the forward speed and the tire slip velocities considered immeasurable for the observer design. The interest of our method is highlighted through both hardware interactive simulations conducted with a human driver in the SHERPA-LAMIH dynamic driving simulator and experimental validation performed using the LAMIH Renault TWINGO experimental vehicle prototype. The obtained results demonstrate the effectiveness and applicability of the proposed estimator under nominal conditions, and then under road friction uncertainties. Finally, the insights that can be gained from our proposed structure can offer valuable conclusions under less restrictive and more realistic assumptions for the interconnected estimation design, robustness, and conservatism, as well as for the practical applicability of the estimation concept.

In future work, various driving situations, such as severe double-lane-change maneuvers for obstacle avoidance, will be investigated. Moreover, the NI-UIO technique will be used together with a fault detection of abnormal driving behavior based on a fault-tolerant controller.

## Figures and Tables

**Table 1:** Robustness to road friction uncertainties in the Satory test track ($\mu_{1}=1$, $\mu_{2}=0.6$, $\mu_{3}=0.4$).

	*r* (%)		$\omega_{f}$ (%)		$\omega_{r}$ (%)		$T_{B_{f}}$ (%)	
	RMSE	NMSE	RMSE	NMSE	RMSE	NMSE	RMSE	NMSE
$\mu_{1}$	1.302	99.73	5.22	97.48	6.05	98.32	5.48	99.69
$\mu_{2}$	1.36	98.19	5.25	97.78	6.65	97.08	5.64	99.62
$\mu_{3}$	1.47	97.94	6.61	97.75	8.09	96.89	5.87	99.72
	$a_{x}$ (%)		$a_{y}$ (%)		δ (%)		$B_{r}$ (%)	
	RMSE	NMSE	RMSE	NMSE	RMSE	NMSE	RMSE	NMSE
$\mu_{1}$	2.4	97.75	1.97	97.02	4.9	96.78	5.99	99.26
$\mu_{2}$	2.91	98.78	2.292	99.76	6.7	92.41	6.12	99.39
$\mu_{3}$	3.27	92.26	2.54	93.77	8.03	94.4	7.82	95.73
	κ (%)		$v_{x}$ (%)		$v_{y}$ (%)		$T_{s}$ (%)	
	RMSE	NMSE	RMSE	NMSE	RMSE	NMSE	RMSE	NMSE
$\mu_{1}$	0.356	98.9	4.72	97.01	5.12	89.9	6.28	98.54
$\mu_{2}$	0.373	98.37	5.23	96.64	5.96	86.62	7.81	98.29
$\mu_{3}$	0.384	97.25	5.86	95.42	7.57	84.61	9.18	92.26

**Table 2:** Vehicle model adequacy evaluation.

	$v_{x}$	$v_{y}$	*r*	$F_{\mathrm{yf}}$	$F_{\mathrm{yr}}$	$F_{\mathrm{xf}}$	$F_{\mathrm{xr}}$	$a_{x}$	$a_{y}$
NMSE(%)	91.67	76.10	90.11	85.22	87.45	79.99	77.13	70.34	89.68
$\frac{RMSE\left(\varepsilon_{x}\right)}{\max\left(\left|x\right|\right)}\left(\%\right)$	1.96	8.67	3.57	5.26	4.47	5.31	8.15	10.98	3.67
$\frac{MEAN\left(\left|\varepsilon_{x}\right|\right)}{\max\left(\left|x\right|\right)}\left(\%\right)$	1.61	5.26	2.58	3.56	3.07	2.83	3.93	8.62	2.42

## Data Availability

Not applicable.

## Appendix A. Vehicle’s Parameters Nomenclature

**Variable**	**Description**
$v_{y}$, $v_{x}$	Lateral and forward velocities
*r*, $a_{x}$, $a_{y}$	Yaw rate and Longitudinal/lateral acceleration
$\omega_{f}$, $\omega_{r}$	Angular velocities of the front and rear wheels
δ, α, λ	Steering angle, side slip angle, and longitudinal slip ratio
$y_{L}$, $\psi_{L}$, κ	Lateral offset and angular displacement, road curvature
$F_{y}$, $F_{x}$, $F_{w}$	Cornering/longitudinal forces and lateral wind force
$F_{a}$, $F_{rr}$	Aerodynamic and rolling resistance forces
$B_{f}$, $B_{r}$, $T_{f}$	Braking torques and engine torque.
*m*, $I_{z}$	Vehicle mass and inertia about the z-axis
$C_{\alpha }$, $C_{\lambda }$	Cornering and longitudinal stiffness parameters
$I_{\omega_{f}}$, $I_{\omega_{r}}$	The wheels’ moment of inertia
$l_{f}$, $l_{r}$	Distances between the C.G. and front and rear axles
$l_{s}$, $l_{w}$	Look-ahead distance and distance of wind force action
$I_{s}$, $R_{s}$	Steering system inertia, column–wheels gear ratio
*R*, $B_{s}$	Wheel radius and damping coefficient.

## Appendix B. Vehicle’s Parameters Values

**Table A1:** Parameters of the Lamih Renault Twingo experimental vehicle prototype.

Parameter	Value	Parameter	Value
m	1210 [kg]	$l_{f}$	1.0065 [m]
$I_{z}$	1520 [kg.m^2^]	$l_{r}$	1.3605 [m]
$C_{f}$	47135 [N/rad]	$l_{s}$	5 [m]
$C_{r}$	56636 [N/rad]	$l_{w}$	0.4 [m]
$C_{\lambda_{f}}$	91165 [N/rad]	*R*	0.2915 [m]
$C_{\lambda_{r}}$	62671 [N/rad]	$I_{s}$	0.02 [kg.m^2^]
$I_{\omega_{f}}$	2.5 [kg.m^2^]	$B_{s}$	5.7295 [Nm/rad/s]
$I_{\omega_{r}}$	2.5 [kg.m^2^]	$R_{s}$	16

## Appendix C

**Proof** **of** **Theorem** **1.** The observer stability was studied by using the following quadratic storage Lyapunov function:

(A1) $$V=e^{T}\underset{P}{\underset{︸}{\left(\begin{array}{cc} \bar{P} & 0\\ 0 & \overset{˘}{P} \end{array}\right)}}e\;P=P^{T}>0$$

Its time derivative is expressed as follows:

(A2) $$\begin{array}{ccc} \dot{V} & = & {\dot{e}}^{T}Pe+e^{T}P\dot{e}\\ & = & e^{T}\left({N_{\eta }}^{T}P+PN_{\eta }\right)e+\Delta_{\xi }^{T}G_{\eta }^{T}Pe+e^{T}PG_{\eta }\Delta_{\xi } \end{array}$$

**Remark** **A1.**
*A more general solution to reduce the conservatives involves deploying more complex structures for the Lyapunov function as a non-quadratic Lyapunov function (NQLF), which is a fuzzy blending of multiple quadratic Lyapunov functions based on the same interconnection structure as the T-S models to be analyzed. The main drawback of NQLF is that the derivative of the Lyapunov function, in the case of continuous systems, involves the appearance of the membership functions’ time derivatives under stability conditions [33]. The problem of the induced conservatism can be partially counterbalanced by the use of the relaxed LMIs conditions— for instance, with some factorizations performed on the weighting functions—by approximating membership functions using staircase or piecewise-linear functions, by introducing additional slack matrices, using some decoupling lemmas such as Tuan’s lemma [37] and Polya’s theorem [38], or Finsler’s lemma [39] and expanding the degree of fuzzy summations [40].*

**Lemma** **A1.**
*For every positive definite matrix G>0, the following property holds:*

(A3)
$$e^{T}PG_{\eta }\Delta_{\xi }+\Delta_{\xi }^{T}G_{\eta }^{T}Pe\leq e^{T}PG_{\eta }GG_{\eta }^{T}Pe+\Delta_{\xi }^{T}G^{-1}\Delta_{\xi }$$

By applying the inequality (A3), replacing the suitable terms, and adding and subtracting the term $\alpha e^{T}Pe$, where α is a positive scalar, the inequality (A2) yields

(A4) $$\begin{array}{cc} \; & \dot{V}<e^{T}\left({N_{\eta }}^{T}P+PN_{\eta }+PG_{\eta }GG_{\eta }^{T}P\right)e+\Delta_{\xi }^{T}G^{-1}\Delta_{\xi }\\ \; & <e^{T}\Psi e-\alpha \underset{V(e)}{\underset{︸}{e^{T}\left(\begin{array}{cc} \bar{P} & 0\\ 0 & \overset{˘}{P} \end{array}\right)e}}+\Delta_{\xi }^{T}G^{-1}\Delta_{\xi }\\ \; & \Psi =\Gamma_{\eta }^{T}P-K_{\eta }C^{T}P+P\Gamma_{\eta }-PK_{\eta }C+PG_{\eta }GG_{\eta }^{T}P+\alpha P \end{array}$$

Now, if Ψ<0, then the time derivative of the Lyapunov function (A4) can be bounded as follows:

(A5) $$\begin{array}{c} \dot{V}\left(t\right)\leq -\alpha V\left(t\right)+\Delta_{\xi }^{T}G^{-1}\Delta_{\xi } \end{array}$$

and the following definition holds. By integrating (A5) over the interval [0,t], we obtain

(A6) $$V\left(t\right)\leq V\left(0\right)e^{-\alpha t}+\frac{G^{-1}}{\alpha }{∥\Delta_{\xi }\left(s\right)∥}_{\infty }^{2}$$

Knowing that V(t) is a Lyapunov function, it can be bounded by $\lambda_{\min}{∥e(t)∥}_{2}^{2}$ and $\lambda_{\max}{∥e(t)∥}_{2}^{2}$, where $\lambda_{\min}$ and $\lambda_{\max}$ are the min and max eigenvalues of the matrix *P*. Under this condition, the state estimation error is reduced to

(A7) $${∥e(t)∥}_{2}\leq \sqrt{\frac{\lambda_{\max}\left(P\right)}{\lambda_{\min}\left(P\right)}}\left({∥e(0)∥}_{2}e^{-\frac{\alpha }{2}t}+\sqrt{\frac{G^{-1}}{\alpha }}{∥\Delta_{\xi }\left(t\right)∥}_{\infty }\right)$$

**Definition** **A1** **([41]).**
*The state estimation error dynamics verify the ISS if there exists a KL function $f_{1}:R^{n}\times R⟶R$, a K function $f_{2}:R⟶R$ such that for each input ξ(t) satisfying ${∥\Delta_{\xi }\left(t\right)∥}_{\infty }<\infty$ and each initial conditions e(0), the trajectory of the error associated to e(0) and Δ(t) satisfies*

(A8)
$${∥e(t)∥}_{2}\leq f_{1}\left({∥e(0)∥}_{2},t\right)+f_{2}\left({∥\Delta_{\xi }\left(t\right)∥}_{\infty }\right)$$

*Hence, when t→∞, the exponential converges to zero, implying the straightforward inequality (A9) from the ISS property*

(A9)
$$\lim_{t\to \infty }{∥e\left(t\right)∥}_{2}<\sqrt{\frac{\lambda_{\max}}{\lambda_{\min}}}\sqrt{\frac{G^{-1}}{\alpha }}\max(∥\Delta_{\xi }\left(t\right){∥}_{\infty })$$

From the boundedness of $\Delta_{\xi }\left(t\right)$ and thanks to Definition (A1), it is shown that the error dynamics (A7) are stable and verify the ISS property from the perturbation term $\Delta_{\xi (t)}$ to the estimation error e(t). Assuming $\lambda_{\min}\left(P\right)\geq 1$ (P>I) and since G can be imposed, minimizing the ISS gain is equivalent to minimizing positive scalars $\eta =diag(\eta_{1},\eta_{2})$ such that

(A10) $$\begin{array}{c} \sqrt{\frac{\lambda_{\max}\left(P\right)}{\lambda_{\min}\left(P\right)\alpha }}\leq \sqrt{\eta }\Rightarrow {\left(\alpha \eta \right)}^{2}I-P^{T}P>0 \end{array}$$

By applying Schur’s complement [42], inequality (A10) can be written as the LMI constraint (27d). The positive quantities $\eta_{1}$ and $\eta_{2}$ are minimized in the objective function given in (27a). This optimization step has been tested intensively, and a similar ISS result was established for LPV systems in [43]. Using the Lyapunov formulation of the ISS property and by exploring the convexity of weighting functions [42], the time-independent LMI conditions of the optimization problem given in (27b)–(27d) can be obtained. Finally, the NI-UIO observer gains are computed from (28) in Theorem 1. □
